# Rise of the king: Gondwanan origins and evolution of megaraptoran dinosaurs

**DOI:** 10.1098/rsos.242238

**Published:** 2025-05-07

**Authors:** Cassius Morrison, Charlie Roger Scherer, Ezekiel V. O’Callaghan, Collin Layton, Colin Boisvert, Mauro Aranciaga Rolando, Leroy Durrant, Pedro Salas, Steven J. R. Allain, Samuel J. L. Gascoigne

**Affiliations:** ^1^ Department of Earth Sciences, University College London, London, UK; ^2^ Fossil Reptiles, Amphibians, and Birds Section, Natural History Museum, London, England, UK; ^3^ Department of Biology, Northern Arizona University, Flagstaff, AZ, USA; ^4^ Department of Neurobiology, University of Pittsburgh, Pittsburgh, PA, USA; ^5^ Carnegie Museum of Natural History, Pittsburgh, PA, USA; ^6^ Oklahoma State University Center for Health Sciences, Tulsa, OK, USA; ^7^ Museo Argentino de Ciencias Naturales Bernardino Rivadavia, Buenos Aires, Argentina; ^8^ Department of Geology and Geophysics, University of Wyoming, Laramie, WY, USA; ^9^ Independent Researcher, Spain; ^10^ Writtle School of Agriculture, Animal and Environmental Sciences, Anglia Ruskin University, Chelmsford, Essex, UK; ^11^ School of Biological Sciences, University of Aberdeen, Aberdeen, UK; ^12^ Department of Biology, University of Oxford, Oxford, UK

**Keywords:** Biogeography, Late Cretaceous, Megaraptora, Tyrannosauroidea

## Abstract

Late Cretaceous Earth was dominated by theropods such as tyrannosauroids and megaraptorans; however, it is unclear how these clades diversified and grew to massive proportions. This study aimed to conduct a biogeographical analysis and test climate as a potential mechanism for the increase in size. We used published phylogenetic matrices with the R package BioGeoBears to test different biogeographical hypotheses for both clades. We mapped body mass (BM) and body length against known climate data to test this potential hypothesis. Continental-scale variance did not drive tyrannosauroid biogeography and instead widespread ancestral populations, sympatric speciation and localized extinctions throughout these clades constricted geographic range. Both patterns were supported by statistical analyses. This biogeographical model also indicates the ancestor of the clade *Tarbosaurus* and *Tyrannosaurus* was present in both Asia and Laramidia, and therefore the ancestor of *Tyrannosaurus* came from Asia. Statistical data illustrated no correlation between Mean Annual Temperature (MAT) and BM but potential climatic shifts may be associated with gigantism in derived megaraptorids and eutyrannosaurians. This biogeographical model implies megaraptorans may have had a cosmopolitan distribution prior to the splitting of Laurasia and Gondwana. Also, gigantism in these clades may be associated with climatic shifts in the Late Cretaceous.

## 1. Introduction

Despite vast amounts of anatomical and phylogenetic analyses over the past decades, some aspects of the evolutionary history of Megaraptora remain elusive. Megaraptora represents a clade of coelurosaurian theropods characterized by long skulls, recurved and proportionally small teeth, highly pneumatic skeletons and long, powerful arms bearing large and sharp manual claws [1–9]. Megaraptorans evolved from small forms in Laurasia (Barremian) [10–12] to large and highly pneumatic species in Gondwana (Turonian-Maastrichtian) [1,3,5,9]. During the Late Cretaceous, this clade became the apex terrestrial predator of western and southern Gondwana after the Cenomanian-Turonian faunal turnover [7,9,13]. However, it is unclear if there was dispersal from Laurasia to Gondwana or if there was a cosmopolitan distribution of Megaraptora prior to the separation of Laurasia and Gondwana [7]. Therefore, despite a concentrated study, the evolutionary history of this clade remains unresolved.

Multiple competing hypotheses exist for the evolutionary trajectory of Megaraptora in the Cretaceous [5,7,9–11]. These competing hypotheses are informed by a fragmentary fossil record, conflicting phylogenetic relationships and wide biogeographic range achieved over a narrow temporal interval. Specifically, the fragmentary fossil record has averted detailed study of this clade’s phylogenetic relationships and prevents an understanding of the true origins and diversification of megaraptorans. This suggests that megaraptorans could be more diverse and more widespread than previously thought. Secondly, conflicting phylogenetic hypotheses for megaraptorans results in differing reconstructions of the biogeographic history. The wide biogeographic range being achieved over a narrow temporal interval may be a result of the aforementioned factors. These inferences represent discrete arguments evidencing the evolutionary history of Megaraptora. However, these findings are insufficient to identify (i) how this clade became globally distributed and (ii) the climatic regimes under which gigantism evolved within the clade.

Related to this matter, the acquisition of gigantic body masses (BM; >1000 kg [14]) and body lengths (BL) are more sparse within Megaraptora and the origin of the more derived megaraptorid clade is not well understood [3,9], which contrasts with tyrannosaurids where gigantism is well documented and observed rapidly after the extinction of Allosauroidea in Laurasia [15,16]. Tyrannosaurs provide a comparative framework for Megaraptorids due to their likely phylogenetic relationship, similar biogeographical origins but differing dispersal trajectories and anatomical evolution. However, there are also competing hypotheses on the evolutionary history and gigantism of *Tyrannosaurus rex*. Phylogenetic evidence has been presented for *T.rex* being derived from an Asian ancestor [17,18]. Meanwhile, Currie *et al*. [19] and geochronological constraints on a new taxon, *T. mcraeensis,* provided evidence for generic evolution in North America [20]. These separate pieces of evidence have left the subject open to interpretation.

Here, we tested two specific hypotheses: (H1) that megaraptorans were widely dispersed prior to the breakup of Laurasia and Gondwana. This hypothesis is supported by multiple fossil records originating from Early Cretaceous Eurasia and Eastern Gondwana [3,9,11]. Secondly, we hypothesized (H2) tyrannosauroid gigantism evolved multiple times in conjunction with temperate climates. This evolutionary trajectory is supported by fossils such as *Yutyrannus* and other tyrannosaurs, such as *T. rex,* being found in cooler climates [21–23]. In other words, the global cooling trend after the Cretaceous Thermal Maximum (CTM) [24] and the extinction of the other apex non-coelurosaurian theropods may have allowed for the evolution of gigantism in megaraptorans and tyrannosauroids. We also hypothesized (H3) that *T. rex* evolved from direct Asian ancestors. This hypothesized descendance is based on the phylogenetic placement of *T. rex* in a clade with other Asian tyrannosaurs [17,18]. Regarding these clades, the origins and dispersal of the tyrannosaurids and megaraptorids have important implications for understanding dinosaurian ecosystem evolution by the end of the Cretaceous.

## 2. Results

### 2.1. Tyrannosauroid and megaraptoran biogeographical history

**Figure 1.**
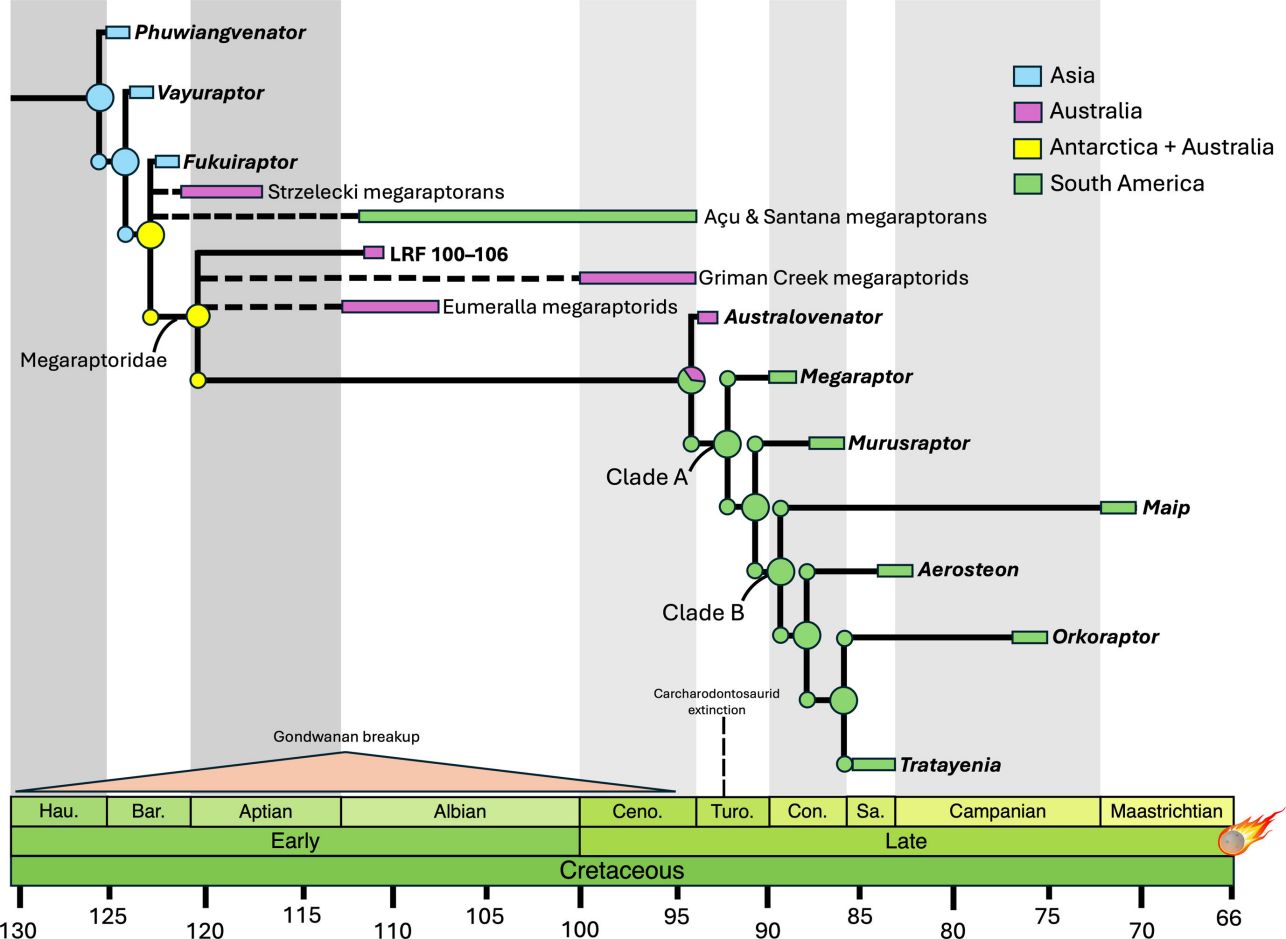
Biogeographic history of Megaraptora with ancestral area reconstructions positioned at each node, highlighting the widespread distribution during early megaraptoran evolution and the rapid evolution of South American Megaraptoridae after the extinction of carcharodontosaurids in the Turonian. Ancestral areas are those estimated by the BAYAREALIKE + J model.

**Figure 2.**
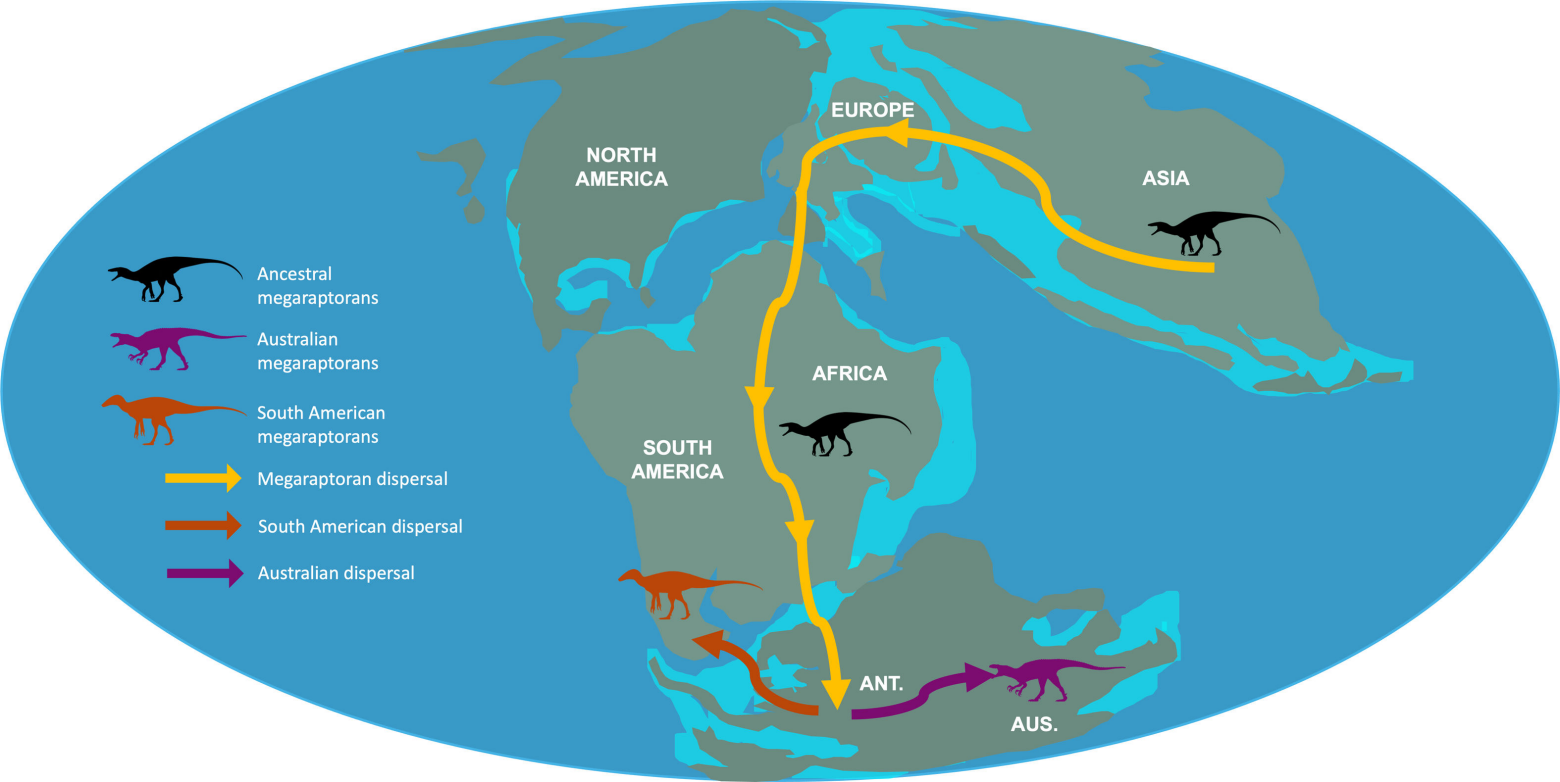
Potential dispersal routes for megaraptorans during the Middle Jurassic–Early Cretaceous (approx. 143 Ma). The dispersal routes were devised using the ancestral area reconstructions in figure 1 and plotting them on a base map redrawn from Scotese [26]. Here, megaraptoran populations disperse via the most-connected regions of geographical areas (i.e. geodispersal). Abbreviations: ANT.: Antarctica; AUS.: Australia. Silhouettes by ‘the funk monk’ and ‘jagged fang designs’ and available from Phylopic.org under a Creative Commons license.

Analyses using the Naish and Cau [25] topology select the BAYAREALIKE+J model as the model that best fits the data, with DEC+J selected as the second-best fit (electronic supplementary material, table S1). This shows that tyrannosauroid biogeography is best described by a wide occurrence of ancestral populations, widespread sympatric speciation as well as eventual localized/regional lineage extinctions which constricted the geographic range of some groups. For megaraptorans, this suggests a wide geographic range in Gondwanan landmasses, including Africa and Antarctica, prior to potential extinction outside South America, although sampling biases in other Gondwanan landmasses may have influenced this interpretation (figures 1 and 2). The lack of support for DIVALIKE models in these analyses suggests that continental-scale vicariance was not a driver of tyrannosauroid biogeography. Analyses using the Aranciaga-Rolando *et al*. [9] topology also select BAYAREALIKE + J as the best-fit model. However, DIVALIKE + J is selected as the second-best-fit model. This suggests that widespread populations, sympatry and extinction explains tyrannosauroid biogeography, but with a much greater contribution from DIVALIKE + J (electronic supplementary material, table S2). This is likely due to the differing placement of Megaraptora between the two topologies, with the topologies of the latter analyses placing megaraptorans as the earliest diverging tyrannosauroids.

Statistical comparison of our models indicates that models incorporating jump dispersal (+J parameter) outperform models without this parameter and that the harsh models outperform most relaxed models (*p* > 0.001; electronic supplementary material, tables S1 and S2). Additionally, the results of analyses performed using the Aranciaga-Rolando *et al*. [9] topology outperform those using the Naish and Cau [25] topology. Therefore, the overall best-fit model is the harsh analysis using the Aranciaga-Rolando *et al.* [9] topology, demonstrating that early divergence, widespread dispersal, subset sympatry and extinction, as well as vicariance of megaraptorans are the most likely processes which explain tyrannosauroid biogeography.

### 2.2. The origin of the genus *Tyrannosaurus*

Analyses of Tyrannosauroidea (excluding Megaraptora) select the DEC + J model as the best fit to the data (AICc wt%: 93; electronic supplementary material, table S3), demonstrating that sympatry, widespread ancestral populations and regional extinctions shaped tyrannosauroid biogeography, even when Megaraptora is excluded from the analyses. *Tyrannosaurus* was recovered as an ancestrally southern Laramidian taxon, with the ancestor of *T. rex* dispersing northward by the Late Maastrichtian, corroborating the results of Dalman *et al*. [20]. However, the model also suggests that the ancestor of *Tyrannosaurus–Tarbosaurus* clade was present in both Asia and Laramidia, suggesting that the direct ancestor of *Tyrannosaurus* dispersed into Laramidia from Asia. This suggests that, regardless of the true age of *T. mcraeensis*, the genus *Tyrannosaurus* originated in Laramidia from an ancestrally Asian taxon that emigrated to North America during the Late Campanian—Early Maastrichtian.

Electronic supplementary material, table S4 uses the assumed sedimentation rates from Dalman *et al*. [20] to find the age for the cf. *Alamosaurus* and the top of the Cretaceous strata were highly variable and difficult to use as an argument for comparison with *T. mcraeensis*. While the morphology and diagnosis of *T. mcraeensis* is detailed, we do not find its diagnosis reliably outside the range of known variation for *T. rex*. Thus, we find the morphology difficult to use for diagnosing the species *T. mcraeensis*.

### 2.3. Climate is not correlated with gigantism

**Figure 3.**
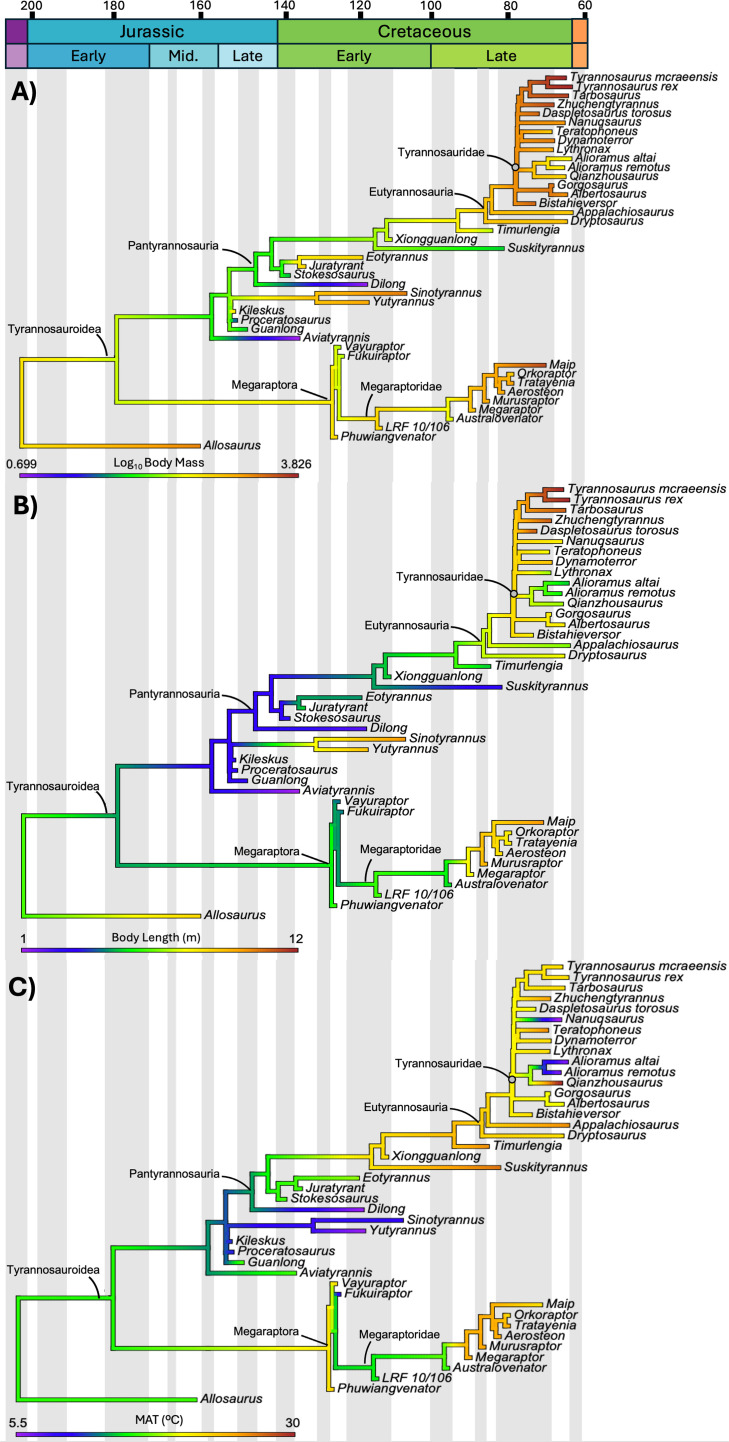
The evolution of body mass (A), body length (B) and habitat mean annual temperature (MAT) (C) across Tyrannosauroidea during the late Early to end-Cretaceous. Body size proxies vary independent of habitat MAT, although both proxies in eutyrannosaurians and megaraptorids show a qualitatively correlated increase after a temperature spike during the Turonian.

Despite occupying diverse mesic and seasonal habitats across both hemispheres, no significant correlations were observed between mean annual temperature (MAT) and BM (R^2^ = 0.303; *p* = 0.05107), even when the data were log-transformed (R^2^ = 0.293; *p* = 0.05954). However, mapping the evolution of MAT across Tyrannosauroidea shows that gigantism likely evolved after a temperature shift (figure 3), after the Cretaceous Thermal Maximum (CTM), as global temperatures cooled into the Campanian-Maastrichtian (electronic supplementary material, figure S1). Body Mass and BL increased in both derived megaraptorids and eutyrannosaurians, indicating that climate shifts may be associated with tyrannosaurid and megaraptoran gigantism (electronic supplementary material, figures S2 and S3). Gigantism is unlikely to be associated with any climatic zones or regimes, however, large size in *Yutyrannus* in a high elevation, cool environment [27] may be an early indicator of success in changing climate regimes, which is otherwise not recognized in the tyrannosaur fossil record. Biases against mountain basins provide limited resolution yet later taxa at high latitudes support *Yutyrannus* as an Early Cretaceous outlier.

## 3. Discussion

Megaraptorans likely originated in Asia, diverging from other tyrannosauroids in the Middle Jurassic (170–165 Ma) with the earliest evidence of the clade approximately 130 Ma (*Phuwiangvenator, Vayuraptor* and *Fukuiraptor*). Populations of megaraptorans then dispersed southward towards Gondwana (Australia and South America) during this period, with analyses suggesting widespread presence in both Australia and Antarctica between 122 and 110 Ma. For example, the presence of non-megaraptorid megaraptorans in South America during the Albian is indicated by material from the Romualdo Member of the Santana Formation [28]. Megaraptoridae became well established during this period (approx. 121 Ma [22,29,30]). Kotevski *et al*. [31] reported on a megaraptorid frontal from early Aptian Australia, corroborating the widespread distribution of megaraptorids during/prior to the Early Cretaceous. This indicates that by the late Early Cretaceous, megaraptorids had achieved a classic Gondwanan distribution (figure 1). Ding *et al*. [32] produced an analysis of the biogeography of non-megaraptoran coelurosaurs, and included a putative tyrannosauroid pubis from Australia. However, Ding *et al*. [32] did not include megaraptoran taxa, but noted the debated status of the group as tyrannosauroids, and a potentially wider southern distribution of the clade. Our analysis supports the results of Ding *et al*. [32], with a western Eurasian dispersal into Africa, and then the rest of the southern continents for megaraptorans, and multiple dispersal events across the Bering Land Bridge between Asia and North America in pantyrannosaurians. This further highlights the significance of Europe as a biogeographic realm.

Bell *et al*. [33] recovered an Early Cretaceous (Barremian–Early Aptian; 130−121 Ma) origin for Megaraptoridae and hypothesized an Australian origin for megaraptorid radiation. Here, we refine the origin of definitive megaraptorids to the early Aptian (approx. 121 Ma) and hypothesize that megaraptorid radiation occurred across Gondwana, with each segment potentially hosting endemic lineages of megaraptorid, although further finds from Cretaceous Australia are needed to test this hypothesis. Additional material from the Bajo Barreal Formation (southern Argentina), which dates to approximately the Cenomanian–Turonian boundary [7,34,35], further corroborates the notion that megaraptorids were widespread during the late Early Cretaceous–early Late Cretaceous and likely dispersed into South America by this time. The large body sizes (7.5−10 m) of the derived South American megaraptorid clade were likely a result of vicariance due to the isolation of this landmass as well as the extinction of large-bodied carcharodontosaurids in the region [9]. The hypothesis that megaraptoran biogeography is best explained by an early acquisition of a Gondwanan distribution during, or prior to, the Early Cretaceous, which was then subjected to Early Cretaceous vicariance, is supported by biogeographic models and numerous indeterminate remains of megaraptorans known from Early Cretaceous deposits in South America and Australia [28,31,36].

The most parsimonious dispersal route for ancestral megaraptoran populations requires passage through Europe into Africa and Antarctica before finally spreading through to South America and Australia (figure 2). The breakup of Gondwana during the Early Cretaceous resulted in vicariance within megaraptora and the establishment of the South American and Australian taxa during the Early Cretaceous (approx. 90−95 Ma). This contradicts previous studies which suggest dispersal from Australia to South America through Antarctica [33]. The passage of megaraptorans to South America is corroborated by material from the Santana [28] and Acu Formations [36]. Similarly, the early Late Cretaceous megaraptoran *Aoniraptor* [36] in South America was recently found as a megaraptorid (M.A.R. *pers. comm.)*, supporting the hypothesis that megaraptorans were widespread during the early Late Cretaceous and the likely role of Early Cretaceous vicariance in driving South American megaraptorid diversification.

Material from the Strzelecki Group (Barremian–Aptian [28,30]), which represents non-megaraptorid Australian taxa from as early as the upper Barremian [9] was recently criticized by Kotevski *et al*. [31]. Nevertheless, these remains were still said to display a combination of features of both non-megaraptorid and megaraptorid megaraptorans [9]. This material was also said to be similar to *Fukuiraptor* and other basal megaraptorans from Asia [9,31], indicating a potential phylogenetic affinity with these taxa which supports the early acquisition for a Gondwanan distribution of Megaraptora, prior to the origin of Megaraptoridae. The combination of Barremian age and diverse morphology of the Strzelecki megaraptorans suggests that both non-megaraptorid and megaraptorid megaraptorans were present in Australia as early as the Late Barremian, further suggesting a widespread distribution of Megaraptora and that an Australian origin for Megaraptoridae is unlikely [31,33].

The presence of megaraptorans in Africa has been suggested by some analyses [37] with the inclusion of the early Late Cretaceous *Bahariasaurus* and *Deltadromeus* as non-megaraptorid megaraptorans. Nevertheless, the megaraptoran affinities for these taxa have been criticized by some authors [8,9,38,39]. If *Bahariasaurus* and *Deltadromeus* are indeed megaraptorans, then the dispersal of the clade through Africa is supported by both fossil evidence and the statistical analyses presented here.

Gigantic BMs in non-avian theropods (>1000 kg; [14,40]) are known to occur in numerous lineages, including abelisauroids, allosauroids, megalosauroids and tyrannosauroids [41–44]. Estimates of BM in tyrannosauroids range from 72 kg to 1414 kg in the proceratosaurids *Guanlong* and *Yutyrannus*, respectively [21,45]. Early diverging pantyrannosaurians such as *Moros intrepidus* have an estimated BM of approximately 72 kg [46]; the mass of non-tyrannosaurid eutyrannosaurians, such as *Dryptosaurus* and *Appalachiosaurus*, have been estimated at BM approximately 1000 kg and approximately 890 kg, respectively [45,47]. Estimates for tyrannosaurids range from approximately 2000 kg for *Albertosaurus* up to approximately 8900 kg in the largest individuals of *T. rex* [45,48]. These estimates demonstrate the evolution of increasing BM in the (Proceratosauridae + Pantyrannosauria) clade and the convergent evolution of gigantism in Proceratosauridae and Eutyrannosauria.

Body length demonstrates a similar pattern, with large (+7 m length) and very large (+10 m length) forms appearing during the course of tyrannosauroid evolution. The same patterns are also apparent in megaraptoran tyrannosauroids [7,9,12], with later-diverging forms having much greater masses and larger estimated body sizes than earlier forms. This pattern is most notable in the derived megaraptorids where body size estimates range from 7−10 metres in length [6,9]. In this sense, during Turonian and Coniacian times, megaraptorids exhibited medium to large sizes (6−7.5 m), while younger species (from Santonian through Maastrichtian) became bigger (8–10 m). This concords with the hypothesis that clade B represents a clade of tyrannosaurid-sized megaraptorids [9]. Unfortunately, too little material of *Maip* is known to produce accurate mass estimates. However, due to the robust nature of the holotype material and the very large size estimate, it is suggested a mass akin to similarly-sized tyrannosaurids (e.g. *Daspletosaurus*; 3−4 tonnes). The increase in BM and BL in eutyrannosaurians and megaraptorids occurs during the same time interval, the early-middle Turonian, suggesting that there was a common, extrinsic factor in both Gondwana and Laurasia, which led to the convergent evolution of gigantism in these clades.

Carcharodontosaurid extinction has long been cited as the driver for body size increases in abelisaurids and tyrannosaurids [9,49,50]. This extinction likely occurred during or after the early-middle Turonian [51] and was potentially linked with increases in global temperatures during the Cenomanian-Turonian Boundary Event (CBTE; 93.9 Ma [52]) caused by an increase in terrestrial volcanism [53,54]. This increase in temperatures was followed by an abrupt decrease in temperatures during the Turonian [55]. These climatic changes may have resulted in conditions that were unsuitable for carcharodontosaurids and may have resulted in their extinction. The extinction of large–very large-bodied carcharodontosaurids would have opened the large-bodied apex predator niche in these ecosystems, allowing mesopredators to occupy this niche. Therefore, the evolution of gigantic BM in tyrannosaurids and megaraptorids was concomitant with, and likely facilitated by, carcharodontosaurid extinction in both Gondwana and Laurasia [9,49,50] during the early-middle Turonian [51] and resulted in the assumption of the apex predator niche by these clades. Our analyses of climate data do not support a correlation between BM and climate in tyrannosauroids, although there appears to be a MAT ‘pulse’ occurring during tyrannosauroid evolution which coincides with the convergent evolution of gigantism. This temperature pulse is concomitant with the CTBE and CTM and may have indirectly driven tyrannosauroid gigantism by contributing to the extinction of carcharodontosaurids and perhaps other non-coelurosaurian theropods.

### 3.1. *Tyrannosaurus* as an endemic North American taxon

Previous studies have suggested that *T. rex* emigrated to North American ecosystems from Asian ecosystems. This hypothesis is based primarily on the closer relationships between *T. rex* and Asian tyrannosaurids, such as *Tarbosaurus* and *Zhuchengtyrannus*, and the more distant relationships with other Laramidian tyrannosaurids, such as *Daspletosaurus* and *Teratophoneus* [17,18,20]. In their description of *T. mcraeensis*, Dalman *et al*. [20] suggested that the genus *Tyrannosaurus* originated in southern Laramidia, due to the proposed Late Campanian–Early Maastrichtian age of the *T. mcraeensis* holotype. Although the exact age of the holotype is disputed, our biogeographical analyses of Tyrannosauridae, particularly the Tyrannosaurini (*sensu* [56]), suggest that this tribe of tyrannosaurines diversified in Asian biotas during the Late Campanian, which is consistent with the hypothesis of previous work [18,57].

Our analyses also indicate that, regardless of the age of *T. mcraeensis*, the genus *Tyrannosaurus* originated in Laramidia, although whether it appeared in the northern or southern portions of the subcontinent is currently unclear, as *Tyrannosaurus* is shown to be widely distributed across Laramidia during the Early Maastrichtian in our analyses. This finding provides further support for the hypothesis that *Tyrannosaurus* was an endemic lineage of tyrannosaurines, which evolved in Laramidian biotas. However, while this supports a Laramidian origin for *Tyrannosaurus*, we do not reject the possibility that the direct ancestor of *Tyrannosaurus* migrated from Asia, as our ancestral area reconstructions estimate an Asiamerican ancestral area for the *Tyrannosaurus–Tarbosaurus* clade, indicating that dispersal of tyrannosaurines from Asia to Laramidia occurred prior to the evolution of either *Tarbosaurus* or *Tyrannosaurus*, likely during the Late Campanian/Early Maastrichtian. Therefore, while *Tyrannosaurus* is likely an endemic, North American genus, the ancestor of the genus dispersed into North American biotas, where it would give rise to *Tyrannosaurus*.

### 3.2. Limitations of the current study

The incomplete fossil record is inherently a limiting factor in this study. This is particularly true within Megaraptora, which are generally poorly represented and mostly known from partial and fragmentary specimens. While many studies have suggested that Megaraptora is within Tyrannosauroidea, debate still exists with alternative phylogenetic trees impacting the potential dispersal routes of Megaraptorans if they have a different placement within Coelurosauria.

Future work with similar methods and different placements of Megaraptorans in relation to Coelurosauria may produce different results, as could additional fossil material from the group that could aid in more accurate taxonomic placement. Compared to most dinosaur clades, new and more complete megaraptoran fossil material spanning the Cretaceous would have a greater impact on their phylogenetic placement and understanding of their origination, diversification and biogeographical relationships due to their current fragmentary nature.

The debate over the relationships of Tyrannosaurini is dependent on fossil remains of highly incomplete taxa such as *T. mcraeensis* and *Zhuchengtyrannus* alongside accurate age dating, which can be limited within sedimentary sequences. Improvements in dating could help elucidate the relationships and origin of *T. rex*. While taxa like *Bahariasaurus* and *Deltadromeus* may have been suggested to have megaraptoran affinities, there is little consensus [8,9,37–39] and more definitive megaraptoran fossils from Africa would improve the biogeographical model proposed in this paper or generate alternative hypotheses. As for climate, we are limited by the availability of climate data for many regions that are often generated by models. While the models cited provide some data, direct proxies for climate data in more regions, formations and localities at the site of the fossil specimens would be ideal, which points to a broader limitation of climate model-based inferences in palaeontology.

## 4. Conclusion

Our biogeographical analysis reveals that Megaraptora had a cosmopolitan distribution prior to the splitting of Laurasia and Gondwana, suggesting Laurasia may yield more megaraptoran fossils with future discoveries and the clade being far more diverse than previously thought. The origin of *T. rex* has been largely debated, but as a genus most likely arose in North America, although its direct ancestors migrated over from Asia, based on our biogeographical model and the likely younger age of *Tyrannosaurus mcraeensis* from sedimentation rate models. Gigantism evolved independently multiple times within the clade Tyrannosauroidea with it being potentially driven by cooler or cooling climates, supporting the idea that disparity within dinosaurian clades was a response to climatic changes. This illustrates the importance of using climate and ecological data to better understand dinosaurian evolution within wider global climatic shifts.

## 5. Material and methods

To identify the spatiotemporal evolutionary origins of megaraptorans and tyrannosauroids, we conducted a biogeographical analysis using published data on megaraptoran and tyrannosauroid occurrences, structured by space and time. Analyses were conducted in R v. 4.3.1 [58], using the BioGeoBEARS package [59], which models various biogeographical processes against a time-calibrated phylogenetic topology. Two recently published megaraptoran phylogenies were tested [9,25] to account for uncertainty in the exact position of Megaraptora within Tyrannosauroidea, and therefore alternative reconstructions of megaraptoran biogeographical history. Geographic and stratigraphic data for all taxa were collected from the primary literature and the topologies were calibrated using the cal3 method [60]. The biogeographic origins of *Tyrannosaurus* were tested using a comprehensive tyrannosaurid topology [61] which accounted for uncertainty in the age of *T. mcraeensis* by using ages of Late Campanian (73 Ma) and Early Maastrichtian (69 Ma) in separate analyses [20].

Taxa were assigned to one or more of the following biogeographical zones: Africa, Appalachia, Asia, Australia, Europe, Laramidia and South America, with each taxon occurring in a maximum of three zones at any point in time. Mannion *et al*. [62] assigned various sauropod taxa, which have yet to be found in a biogeographical zone with ‘?’ in their geographic range files and developed ‘rules’ for such an assignment, in an attempt to account for uneven sampling for sauropod taxa globally. These ‘rules’ primarily account for the paucity of terrestrial deposits for certain time intervals in particular geographic regions (e.g. Middle Jurassic, North America; Early Cretaceous, Indo-Madagascar; latest Cretaceous, Australia). This approach was employed here, where taxa are assigned ‘?’ for the latest Cretaceous (Campanian-Maastrichtian) of Australia. The possibilities of megaraptorans occurring in Africa and Antarctica are accounted for by assigning values of ‘?’ for the presence/absence of these taxa in this region, due to the relative paucity of sampling of sedimentary strata here. This approach allowed us to account for sampling biases in these geographic regions and more rigorously test megaraptoran biogeographic history. Tyrannosauroid body size evolution was reconstructed using published mass and length estimates for all valid tyrannosaurid taxa and plotted against a comprehensive tyrannosauroid topology, grafting together the results of Carr *et al*. [61] and Aranciaga-Rolando *et al*. [9]. This allowed us to trace the progression of BM and BL across all tyrannosauroids during the Cretaceous and assess the effect of climate on the evolution of gigantism. Please see the electronic supplementary material, text for the full methodology.

## Data Availability

The datasets used and/or analyzed during the current study are available as part of the supplementary information [63].
